# Vascular Smooth Muscle Cell Plasticity in Atherosclerosis: Mechanisms, Recent Advances, and Therapeutic Implications

**DOI:** 10.31083/RCM50363

**Published:** 2026-08-05

**Authors:** Tianhan Li, Xinyao Niu, Liaoxun Lu, Yinming Liang, Lichen Zhang

**Affiliations:** ^1^School of Basic Medicine, Henan Medical University, 453003 Xinxiang, Henan, China; ^2^School of Medical Technology, Henan Medical University, 453003 Xinxiang, Henan, China

**Keywords:** atherosclerosis, vascular smooth muscle cells, phenotypic switching, single-cell omics, targeted therapy

## Abstract

Atherosclerosis, the fundamental pathological basis of most cardiovascular diseases which remain the leading cause of global mortality, is driven by both lipid accumulation and dynamic cellular reprogramming within the vessel wall. Central to this process is the remarkable phenotypic plasticity of vascular smooth muscle cells (VSMCs). Far from being terminally differentiated, VSMCs undergo profound transitions from a contractile state to diverse states, including synthetic, macrophage-like, foam cell-like, and fibroblast-like states, which critically influence plaque formation, stability, and rupture. This review synthesizes the multilayered molecular mechanisms governing VSMC plasticity, encompassing transcriptional networks, epigenetic reprogramming, and microenvironmental cues. Moreover, this review highlights recent breakthroughs enabled by single-cell omics and lineage tracing, that have revealed unprecedented heterogeneity and clonal expansion of VSMCs within atherosclerotic lesions. Furthermore, we explore the translational potential of targeting VSMC plasticity, and discuss emerging strategies, including phenotype-specific modulation, immunotherapy, nanomedicine, and senotherapeutics. Finally, we outline future directions focused on dynamic regulatory networks, spatial pathophysiology, and the integration of aging biology to advance precision medicine in atherosclerosis. In summary, VSMC phenotypic plasticity is a core mechanism underlying the initiation and progression of atherosclerosis. Precise modulation of this process holds promise for overcoming current therapeutic limitations and driving a paradigm shift toward mechanism-guided personalized therapy for cardiovascular diseases.

## 1. Introduction

Atherosclerosis, a chronic inflammatory disease of the arterial wall [1], remains the leading global cause of cardiovascular morbidity and premature mortality despite decades of therapeutic advancements. While historically considered a lipid-driven disorder, atherosclerosis is now recognized as a chronic inflammatory disease wherein immune responses and cellular plasticity are equally critical [2]. Within this complex pathological milieu, vascular smooth muscle cells (VSMCs) have emerged as central players that extend far beyond their traditional contractile functions. These cells exhibit remarkable phenotypic plasticity, enabling them to adopt diverse identities in response to local environmental cues [3,4]. This fundamental biological process not only profoundly influences atherosclerotic plaque development, progression, and clinical complications [5], but also represents a crucial therapeutic target; understanding its regulation of plaque fate is key to developing novel strategies against this pervasive cardiovascular disease.

The concept of VSMC plasticity challenges earlier paradigms that viewed these cells as terminally differentiated contractile elements. Contemporary research utilizing advanced lineage-tracing techniques and single-cell technologies has revealed that VSMCs can undergo substantial phenotypic modulation, contributing to multiple aspects of plaque biology [6]. In healthy arteries, VSMCs reside primarily in the medial layer, where they regulate vascular tone and maintain structural integrity through extracellular matrix production [5,7]. During atherogenesis, however, they migrate to the intima, proliferate, and transform into various phenotypes that can either stabilize plaques through fibrous cap formation or destabilize them by promoting inflammation, calcification, and necrotic core expansion [8,9]. Notably, VSMCs dysfunction extends beyond atherosclerosis; their pathological hyperreactivity is also a central mechanism in coronary artery spasm, an acute vascular event that can lead to variant angina, myocardial infarction, and even sudden cardiac death, highlighting the pivotal role of VSMCs in a broader spectrum of cardiovascular pathophysiology [10].

This review synthesizes current knowledge on the fundamental mechanisms driving VSMC plasticity, then showcase recent technological advances redefining our understanding, and finally critically appraise the therapeutic strategies emerging from these insights.

## 2. Basic Mechanisms Underlying VSMC Plasticity

Under physiological conditions, VSMCs within the arterial media predominantly adopt a differentiated, quiescent contractile phenotype, which sustains vasomotor homeostasis. However, in response to cues from the atherosclerotic pathological microenvironment, VSMCs undergo phenotypic switching: they downregulate contractile phenotype markers and transdifferentiate into a synthetic phenotype, alongside heterogeneous states (e.g., macrophage-like and foam cell-like VSMCs).

**Fig. 1.**
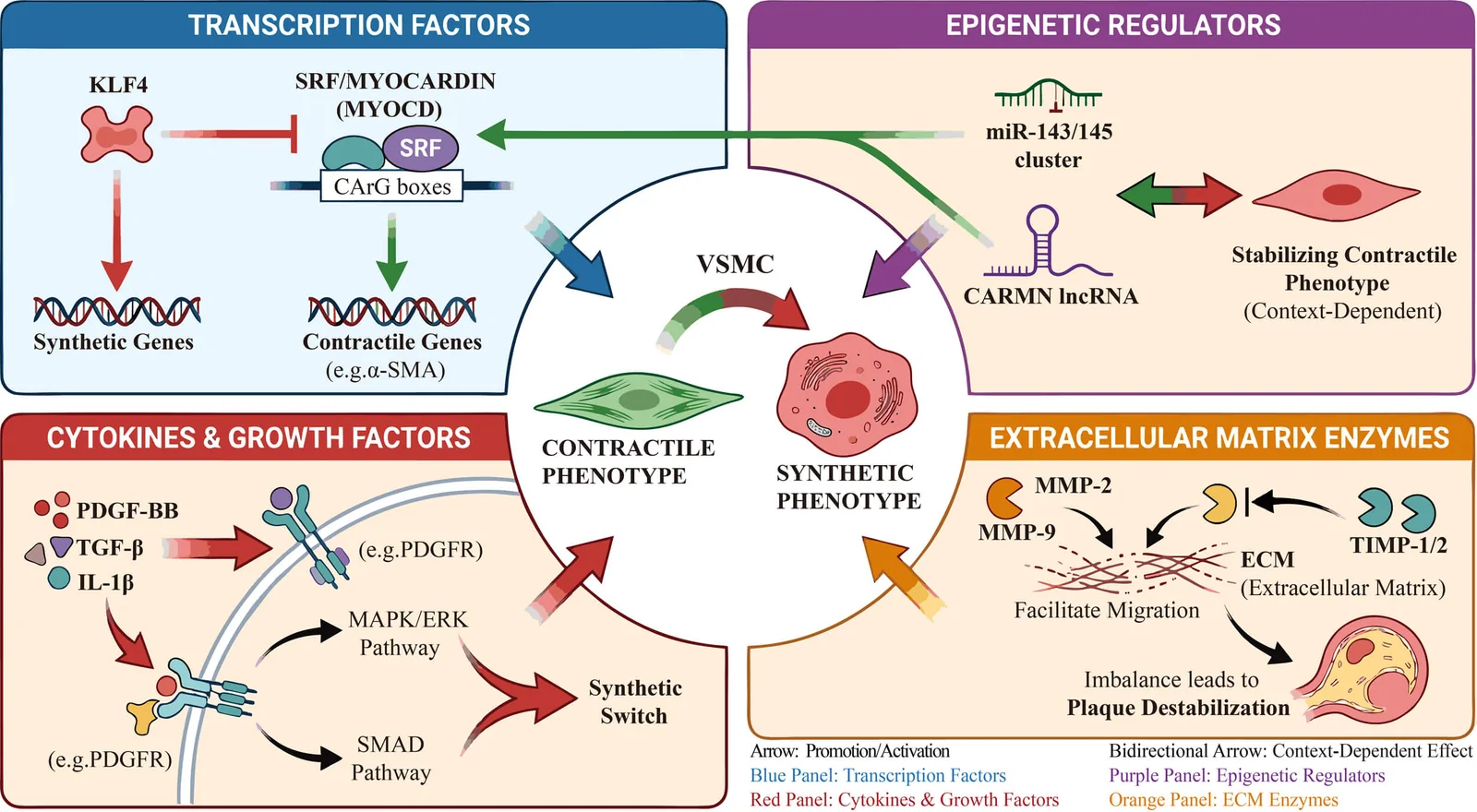
**Multidimensional regulation of vascular smooth muscle cell (VSMC) phenotypic plasticity**. This figure illustrates the core regulatory network governing VSMC phenotypic plasticity. Under stimulation from the atherosclerotic microenvironment, VSMCs transition from a contractile to a synthetic phenotype through coordinated regulation by transcription factors (e.g., krüppel‑like factor 4 (KLF4), serum response factor (SRF), myocardin), epigenetic modifiers (e.g., miR-143/145, CARMN lncRNA), extracellular matrix enzymes (e.g., matrix metalloproteinase-2 (MMP-2), MMP-9), and cytokine signaling pathways (e.g., platelet-derived growth factor BB (PDGF-BB), transforming growth factor‑β (TGF-β)). Interactions among these modules determine VSMC fate, influencing plaque formation, stability, and progression. IL-1β, interleukin-1β; SMAD, small mothers against decapentaplegic; ECM, extracellular matrix; TIMP-1/2, tissue inhibitor of metalloproteinase; α-SMA, alpha-smooth muscle actin; PDGFR, platelet-derived growth factor receptor; CARMN lncRNA, cardiac mesoderm enhancer-associated non-coding RNA.

This schematic (Fig. 1) delineates the core regulatory pathways governing this process, encompassing multidimensional mechanisms including transcriptional regulation, epigenetic modification, cytokine signaling, and extracellular matrix (ECM) crosstalk. These regulatory modules act synergistically or antagonistically to modulate VSMC phenotypic plasticity, thereby shaping the initiation, progression, and evolution of atherosclerotic plaques. Deciphering this regulatory network establishes a foundational theoretical framework for the development of atherosclerosis therapeutic and preventive strategies targeting VSMC phenotypic switching.

### 2.1 Molecular Pathways Driving Phenotypic Switching

The transition of VSMCs from a contractile to a synthetic phenotype involves complex signaling networks and transcriptional reprogramming. Key mediators include platelet‑derived growth factor (PDGF), transforming growth factor‑β (TGF‑β), and various inflammatory cytokines, which activate specific intracellular cascades [11,12]. Platelet-Derived Growth Factor BB (PDGF‑BB) is a critical inducer of the shift from a healthy contractile state to a pathological synthetic phenotype, while thymosin β4 can limit this conversion by regulating the endocytosis and degradation of PDGF‑BB signaling [13].

Furthermore, the *SLC44A2* gene maintains the contractile phenotype of VSMCs by interacting with neuropilin-1 (NRP1) and integrin subunit beta 3 (ITGB3) to activate the TGF‑β/small mothers against decapentaplegic (SMAD) pathway; inhibition of *SLC44A2* promotes the switch toward a synthetic phenotype [14]. In the context of progeria, progerin‑induced VSMC dysfunction and death lead to localized upregulation of TGF‑β1 in the aorta, which drives endothelial‑to‑mesenchymal transition (EndMT) via the SMAD3 pathway, thereby promoting atherosclerotic plaque formation and instability [15]. TGF‑β signaling drives VSMCs toward an inflammatory phenotypic switch, migration into the intima, and proliferation, whereas factors such as angiogenic factor with G patch and FHA domain 1 (AGGF1) can stabilize the VSMC phenotype by inhibiting the maturation and activation of TGF‑β [16].

Krüppel‑like factor 4 (KLF4) has been identified as a key transcription factor that represses contractile gene expression and is required for VSMC phenotypic switching, while also promoting VSMC proliferation and migration [17,18,19,20]. The RNA‑binding protein RNA-binding motif protein 24 (RBM24) drives VSMC phenotypic conversion by stabilizing Janus kinase 2 (JAK2) mRNA and activating the JAK2/Signal transducer and activator of transcription 3 (STAT3)/KLF4 axis [21]. Recent studies further elucidate upstream regulators of KLF4: the cytoskeleton‑associated protein Nexilin (NEXN) maintains the contractile phenotype by suppressing the endoplasmic reticulum stress/activating transcription factor 4 (ATF4)/KLF4 axis; downregulation of NEXN relieves this inhibition, thereby promoting the synthetic switch and neointimal hyperplasia [22]. Additionally, Suppressor of variegation 3-9 homolog 1 (SUV39H1) acts as a critical epigenetic regulator induced by PDGF, promoting KLF4 expression through increased repressive histone mark H3K9me3 and chromatin accessibility remodeling, thereby driving VSMC dedifferentiation and intimal hyperplasia [23].

Similarly, serum response factor (SRF) and its co‑activators play dual roles: maintaining the contractile phenotype under physiological conditions while promoting the synthetic switch in pathological states [12,24,25]. Specifically, loss of SUMO-specific protease 1 (SENP1) promotes SUMOylation of SRF at lysine 143, shifting the SRF‑myocardin complex toward an SRF‑ETS Like-1 protein (ELK1) complex, which markedly exacerbates pathological phenotypic switching and vascular remodeling; targeting this axis (e.g., with AZD6244) may offer a novel therapeutic strategy for related cardiovascular diseases [26]. Further research shows that vascular injury or PDGF stimulation activates ELK1, which directly induces expression of *Staphylococcal nuclease and Tudor domain containing 1* (*SND1*). Upregulated SND1 binds SRF, competitively displaces myocardin (MYOCD), and recruits histone acetyltransferase lysine acetyltransferase 2B (KAT2B) to enhance chromatin accessibility, thereby cooperatively activating transcription of proliferation‑ and migration‑related genes and forming a positive feedback ELK1/SND1/SRF loop that drives VSMC phenotypic switching and neointimal hyperplasia [27]. Furthermore, the mitogen-activated protein kinase/extracellular signal-regulated kinase (MAPK/ERK) signaling pathway serves as a critical hub connecting extracellular stimuli with VSMC phenotypic responses. This pathway is widely activated under infectious and inflammatory conditions and participates in VSMC phenotypic switching by regulating cell proliferation, differentiation, and apoptosis [28]. Within the atherosclerotic microenvironment, inflammatory cytokines and mechanical stress activate the MAPK cascade, which in turn promotes the transition of VSMCs toward a synthetic phenotype and exacerbates intra-plaque inflammation through the phosphorylation of downstream transcription factors such as ELK1 [29,30,31].

Moreover, cellular energy metabolism serves as a fundamental driver of VSMC phenotype [32,33]. For instance, the mitochondrial protein Prohibitin 2 (PHB2) maintains the contractile phenotype and normal energy metabolism in VSMCs by directly binding the splicing factor Heterogeneous nuclear ribonucleoprotein A1 (hnRNPA1) to suppress generation of the pro‑glycolytic isoform Pyruvate kinase M2 (PKM2); loss of PHB2 function reprograms cellular metabolism toward glycolysis, promoting the transition to a synthetic phenotype [34]. Lipid metabolism is also a key driver of VSMC phenotypic switching. VSMCs can acquire a foam cell-like phenotype through the uptake of oxidized low-density lipoprotein (ox-LDL), a process involving the reprogramming of lipid metabolism-related genes and coordinated regulation by multiple transcription factors and signaling pathways [35,36]. For instance, the natural product paeoniflorin significantly inhibits ox-LDL-induced VSMC foam cell formation by upregulating the expression of ABCA1, a key protein involved in cholesterol efflux, thereby blocking pathological phenotypic switching, further highlighting the critical role of lipid metabolism regulation in maintaining VSMC homeostasis [37]. Another core pathway closely associated with metabolic reprogramming is the phosphatidylinositol 3-kinase (PI3K)/protein kinase B (AKT)/mammalian target of rapamycin (mTOR) signaling axis. This pathway is widely involved in metabolic regulation, autophagy, and inflammatory responses in both immune and vascular cells [38,39]. In VSMCs, mTOR activation promotes synthetic phenotypic switching and proliferation while inhibiting autophagy, thereby exacerbating plaque progression. Targeting this pathway or its upstream regulators, such as PI3Kβ, may represent a novel strategy to intervene in pathological VSMC switching [40].

In the regulatory network governing VSMC plasticity, epigenetic modifications occupy a central role, dynamically modulating gene expression patterns during phenotypic switching [41,42]. For instance, histone acetyltransferases p300 and CREB-binding protein (CBP) coordinate opposing chromatin remodeling programs—promoting differentiation in cooperation with Ten-eleven translocation 2 (TET2), while facilitating dedifferentiation alongside histone deacetylases (HDACs)—thereby playing critical yet opposing roles in VSMC phenotypic modulation [43]. Furthermore, the long non-coding RNA (lncRNA) CARMN can directly interact with SRF to enhance its promoter occupancy, thereby regulating VSMC plasticity in atherosclerosis [44]. The circular RNA (circRNA) hsa_circ_0001402 functions as a molecular sponge for miR-183-5p, upregulating FKBPL and BECN1 expression to simultaneously inhibit VSMC proliferation and migration while promoting autophagy, thereby mitigating neointimal hyperplasia [45]. At the microRNA (miRNA) level, earlier studies established that miR-143 and miR-145 are essential for maintaining the contractile phenotype of VSMCs, and their downregulation drives phenotypic switching [46,47]. Conversely, aging promotes the acquisition of a senescence-associated secretory phenotype (SASP) in VSMCs via the tumor protein p53 (p53)/miR-1204/myosin light chain kinase (MYLK) axis, exacerbating vascular inflammation and contributing to the development of aortic aneurysm and dissection [48]. Together, these mechanisms reveal a complex epigenetic regulatory network that extends beyond protein-coding genes [49,50]. This regulatory nexus is critically implicated in major vascular remodeling diseases; for instance, in aortic aneurysms, distinct miRNA expression profiles have been shown to differentially drive VSMCs toward pathological phenotypes such as apoptosis, calcification, or fibrosis, underscoring their potential as disease-specific biomarkers and therapeutic targets [51]. Notably, recent functional screening studies provide further direct evidence for this regulatory network by identifying seven novel miRNAs—miR-323a-3p, miR-449b-5p, miR-491-3p, miR-892b, miR-1827, miR-4774-3p, and miR-5681b—that effectively inhibit VSMC proliferation and migration with minimal impact on endothelial cells, highlighting their therapeutic potential [52].

### 2.2 Environmental Cues and Cellular Interactions

The atherosclerotic microenvironment harbors multiple stimuli capable of driving VSMC plasticity. Ox-LDL, pro-inflammatory cytokines, and hemodynamic forces act in concert to promote phenotypic modulation of these cells [6,53,54]. For instance, Ox‑LDL promotes VSMC phenotypic switching to a synthetic state and increases cell migration through miR‑4463 downregulation and subsequent c-Jun N-terminal kinase (JNK)/ERK pathway activation [55]. Hemodynamic forces regulate endothelial KLF4, which in turn governs the production of the paracrine factor Bone morphogenetic protein 6 (BMP6), thereby critically and indirectly dictating VSMC fate toward a pathological osteogenic phenotype [56]. VSMCs engage in extensive crosstalk with neighboring cells, including endothelial cells, macrophages, and T cells, via paracrine signaling and direct contact [57,58,59]. Within this network, activated macrophages play a pivotal driving role: by secreting a plethora of inflammatory factors (e.g., interleukin-1β (IL-1β), tumor necrosis factor-α (TNF-α)), they create a pathological microenvironment that facilitates the “osteogenic-like transdifferentiation” of VSMCs, activating their Runt-related transcription factor 2 (RUNX2) signaling pathway and ultimately leading to vascular calcification [60]. Certain multi-target regulators, such as Ganoderma lucidum spore powder, can interrupt this vicious cycle by simultaneously suppressing macrophage inflammation and directly blocking osteogenic differentiation of VSMCs [61]. Conversely, in diseases like aortic dissection, macrophages can exert a protective, homeostatic function by efficiently clearing apoptotic VSMCs via EGF-like repeats and discoidin I-like domains 3 (EDIL3)-mediated efferocytosis [62]. These multifaceted interactions form reinforcing feedback loops that further exacerbate phenotypic alterations in VSMCs.

VSMCs also contribute to adaptive immunity within plaques by participating in the formation of artery tertiary lymphoid organs (ATLOs) in the adventitia [63,64]. In this capacity, they adopt lymphoid tissue organizer-like phenotypes, expressing chemokines such as C-C motif chemokine ligand 19 (CCL19), C-C motif chemokine ligand 21 (CCL21), and C-X-C motif chemokine ligand 13 (CXCL13) that recruit and organize T- and B-cells. This previously underappreciated function highlights the immunological roles of VSMCs and their integration into broader inflammatory networks within the atherosclerotic artery [60,65,66]. A recent study utilizing single-cell and spatial transcriptomic technologies has identified plaque tertiary lymphoid organs (PTLOs) in human carotid artery plaques, revealing their role in promoting B-cell clonal expansion and antibody production, thereby enhancing adaptive immune responses and contributing to plaque instability [67]. Table 1 summarizes the critical molecules that modulate VSMC plasticity during the pathogenesis of atherosclerosis.

**Table 1.** **Key molecular regulators of VSMC plasticity in atherosclerosis**.

Regulator type	Specific examples	Primary function in VSMC plasticity	Net effect on plaque
Transcription Factors	KLF4	Repress contractile genes, promote synthetic phenotype	Increased proliferation/migration
	SRF, MYOCD	Promote contractile genes, maintain the contractile phenotype	MYOCD loss promotes plaque progression
Epigenetic Regulators	CARMN lncRNA, miR-143/145	Modulate gene expression via SRF interaction or mRNA targeting	Direction dependent on context
Cytokines/Growth Factors	PDGF-BB, TGF-β, IL-1β	Activate intracellular signaling pathways	Promote synthetic switch
Extracellular Matrix Enzymes	MMP-2, MMP-9, TIMP-1/2	Remodel microenvironment, facilitate migration	Destabilization if unbalanced

This table summarizes representative regulatory factors from four categories, including transcription factors, epigenetic regulators, cytokines/growth factors, and ECM enzymes, which control VSMC phenotypic switching. For each type, specific examples are provided, along with their primary functions in modulating VSMC plasticity and the net effect on atherosclerotic plaque formation or stability. MYOCD, myocardin.

## 3. Recent Advances in Understanding VSMC Plasticity

### 3.1 Single-Cell Technologies Revealing Unprecedented Heterogeneity

The application of single-cell RNA sequencing (scRNA-seq) and genetic fate-mapping has revolutionized our understanding of VSMC contributions to atherosclerosis. These technologies have uncovered an extraordinary diversity of VSMC-derived cell states within plaques, challenging the traditional binary view of contractile versus synthetic phenotypes. Recent studies have identified multiple distinct clusters, including macrophage-like, fibroblast-like, osteochondrogenic, and mesenchymal stem cell-like populations, all originating from VSMCs [6,17,68,69].

Notably, spatial transcriptomics has enabled researchers to map these diverse cell states within specific plaque microenvironments. This approach has revealed that VSMC-derived cells localize to distinct regions—synthetic phenotypes often occupy the fibrous cap, while osteochondrogenic cells associate with calcified areas, and macrophage-like cells accumulate in inflammatory zones. This spatial organization suggests specialized functions for different VSMC derivatives in plaque pathology [5,8,64,70,71].

### 3.2 Novel Biomarkers and Lineage-Specific Markers

Recent studies have identified new markers capable of distinguishing VSMCs subpopulations, holding potential diagnostic and therapeutic value. Fibroblast activation protein (FAP) has been identified as a specific marker for modulated VSMCs within the intima of human atherosclerotic plaques [72,73]. Immunohistochemical studies revealed that FAP is not expressed in normal coronary arteries but is expressed in atherosclerotic lesions, with its expression level increasing with plaque progression; its distribution pattern also varies across different lesion types [74]. Genetic lineage tracing experiments conducted in mice confirmed that FAP^+^ cells originate from medial VSMCs, while non-invasive positron emission tomography (PET) imaging in patients demonstrated concentrated uptake of FAP in atherosclerotic lesion areas [72,75], highlighting its translational potential. Functional studies indicate that FAP promotes the formation and instability of atherosclerotic plaques by degrading extracellular matrix, particularly collagen [67].

Another significant advancement is the finding that clonal expansion of a small number of VSMCs contributes substantially to the increase in plaque cellularity [76]. Early studies using X-chromosome inactivation analysis by Benditt et al. [77] initially indicated the clonal nature of plaque VSMCs. Subsequent Polymerase Chain Reaction (PCR) -based analyses of X-linked genes further validated this phenomenon [78,79]. This finding has been reinforced by advanced lineage tracing techniques: Feil et al. [80] observed single-color labeling of plaque VSMCs in mice, supporting oligoclonal expansion; Chappell et al. [81] confirmed that most plaque VSMCs originate from 1 to 2 precursor cells; and Jacobsen et al. [82] found that under prolonged high-fat diet conditions, plaque VSMCs in mice were predominantly derived from a single source. These studies collectively suggest that atherosclerotic lesions are formed through extensive proliferation and phenotypic differentiation of relatively few precursor cells [83]. As summarized in a review by Luo et al. [83], both human sample studies and mouse experiments support the monoclonal or oligoclonal expansion characteristic of VSMCs. This clonal property is of great significance for a deeper understanding of the mechanisms underlying the initiation and development of atherosclerotic plaques. Research by Worssam indicates that oligoclonal expansion stems from low-frequency proliferative activation of a small subset of VSMCs, and that ROCK1 protein levels are significantly lower in smooth muscle cell-specific antigen 1 (SCA1^+^) VSMCs compared to SCA1^–^ cells [84]. Furthermore, Lin et al. [85] emphasize that this clonal expansion is not an isolated phenomenon but a common feature in cardiovascular diseases, and that its phenotypic differentiation is finely regulated by the local microenvironment. Additionally, Azar et al. [86] point out that clonal expansion directly influences the phenotypic switching process of VSMCs, which is associated with plaque stability.

### 3.3 The Emerging Role of VSMC Senescence

In atherosclerosis research, cellular senescence has been identified as a key factor influencing VSMCs behavior [87]. Senescent VSMCs exhibit a pro-inflammatory secretory phenotype, releasing cytokines such as IL-6 and TNF-α, which exacerbate local inflammation and tissue damage [88,89]. Concurrently, their diminished regenerative capacity predisposes to impaired vascular repair and increases the risk of post-interventional restenosis, particularly evident in elderly patients [90].

Recent studies have further unraveled the complex regulatory mechanisms and therapeutic potential of VSMC senescence. On one hand, regarding molecular mechanisms, while replicative senescence and stress-induced senescence in VSMCs share a general decline in histone modification levels such as H3K9me3 and H3K4me3, they exhibit significant differences in their epigenetic landscape distribution and transcriptomic profiles, revealing senescence type-dependent epigenetic reprogramming [91]. The mitochondrial protein TNF receptor-associated protein 1 (TRAP1) was found to be upregulated in atherosclerotic plaques. It promotes lactate accumulation by enhancing aerobic glycolysis, which in turn inhibits the histone delactylase HDAC3, leading to increased lactylation of histone H4 at lysine 12 (H4K12la). This modification activates the expression of SASP genes, thereby driving cellular senescence and atherosclerosis progression [92]. Notably, this paradigm extends beyond senescence to other pathological endpoints of VSMC plasticity.

For instance, in vascular calcification, a process often associated with aged and diseased vessels, the orphan nuclear receptor nuclear receptor subfamily 4 group A member 3 (NR4A3) is upregulated and promotes glycolysis and lactate production. This subsequently increases histone H3 lactylation at lysine 18 (H3K18la), which activates pro-calcific gene expression [93]. Together, these studies underscore that glycolysis-driven histone lactylation serves as a fundamental metabolome-epigenome signaling axis that can steer VSMCs toward diverse dysfunctional states, including senescence and calcification, depending on the cellular context and upstream triggers. Simultaneously, another important protective axis—the Sirtuin 6 (SIRT6)–Carboxyl terminus of Hsc70-interacting protein (CHIP)–telomere maintenance axis, has been identified. Research shows that the deacetylase SIRT6 is significantly downregulated at the protein level in plaque VSMCs, and its stability is regulated by the ubiquitin ligase CHIP. SIRT6 maintains telomere integrity by deacetylating telomeric histone H3K9. Loss of its activity leads to telomere damage, thereby inducing VSMC senescence and promoting atherosclerosis [94].

On the other hand, concerning pathological significance and therapeutic concepts, growing evidence suggests that senescence should not be viewed merely as a passive terminal state, but rather as an active and plastic cellular phenotype. It may even serve as an intermediate state facilitating the transition of VSMCs into other pathological phenotypes, such as fibroblast-like or fibrochondrocyte-like cells [95,96]. A recent study found that cellular senescence can also inhibit TGF-β signaling by activating the Stimulator of interferon genes (STING)/interferon regulatory factor 3 (IRF3) pathway, thereby significantly impairing the ability of VSMCs to re-differentiate into a contractile phenotype. This may further exacerbate disease progression from the perspective of “cellular phenotype locking” [97]. The persistent SASP of senescent cells creates a chronic pro-inflammatory microenvironment, compromising plaque stability and promoting disease development [98].

Based on this understanding, targeting senescent cells has emerged as a highly promising therapeutic strategy. Beyond specific interventions like genetic knockout or proteolysis-targeting chimera (PROTAC)-mediated TRAP1 degradation, senolytic agents (e.g., dasatinib, quercetin, fisetin) and senomorphic agents (e.g., metformin, statins, dipeptidyl peptidase 4 (DPP4) inhibitors) have demonstrated potential in preclinical models to eliminate senescent cells or suppress their detrimental secretions, thereby reducing plaque burden and improving vascular function [99,100,101,102,103,104]. For instance, senescent VSMCs can drive a unique “senohemostasis” phenotype, centered on complement and coagulation factors, by overexpressing DPP4, directly leading to plaque destabilization. Inhibiting DPP4 can simultaneously clear senescent cells and suppress their harmful secretions, thereby improving plaque structure [88]. Additionally, modulating the function of transcription factors such as SRY-box transcription factor 9 (Sox9) to regulate the composition and stiffness of the extracellular matrix is viewed as a potential direction for alleviating vascular stiffening and senescence [105]. In support of this approach, studies have identified CCAAT/enhancer-binding protein alpha (C/EBPα) as a key upstream regulator of Sox9 in vascular calcification. C/EBPα expression is upregulated during calcification and promotes osteochondrogenic transdifferentiation of VSMCs via Sox9 activation, suggesting that targeting the C/EBPα–Sox9 axis could be a viable strategy to mitigate vascular calcification and stiffness [106].

## 4. Commonly Used Mouse Models in Related Research

In atherosclerosis research, mouse models have become a central tool for deciphering the regulatory mechanisms of VSMCs phenotypic plasticity and for validating the efficacy of targeted therapeutic strategies, owing to their well-defined genetic backgrounds, short reproductive cycles, and suitability for precise genetic manipulation. Models based on genetic modification and lineage tracing technologies enable specific labeling of VSMCs, regulation of key gene expression, or simulation of pathological microenvironments, thereby providing indispensable experimental support for tracking VSMC fate transitions and elucidating their heterogeneity and clonal expansion characteristics.

The mouse strains summarized in Table 2 (Ref. [19,107,108,109,110,111,112]) include both gene knockout models and Cre-loxP-mediated conditional gene editing/lineage tracing models, each suited for distinct research purposes: Gene knockout models (e.g.,* Klf4*-KO,* Notch3*-KO) are primarily employed to elucidate the essential functions of specific genes in maintaining and modulating VSMC phenotypes, revealing the physiological and pathological roles of transcription factors and key signaling molecules. In contrast, VSMC-specific Cre driver mice (e.g., *SM22α*-Cre, *Myh11*-CreER) utilize spatiotemporally specific promoters to drive Cre recombinase expression, enabling conditional knockout, knock-in, or lineage labeling of target genes within VSMCs. The use of these models has not only advanced the mechanistic understanding of VSMC plasticity but also provided standardized experimental platforms for the preclinical validation of novel therapeutic targets, thereby facilitating the translation of fundamental atherosclerosis research into clinical applications.

**Table 2.** **Mouse models for genetic manipulation and lineage tracing of VSMC**.

Mouse Strain	Advantages	Limitations	References
*Klf4*-KO	Elucidates the crucial role of Klf4 in maintaining the contractile phenotype of VSMCs; precise.	Requires combination with Cre driver lines; phenotype may be influenced by the microenvironment.	[19]
*Oct4*-KO	Validates the potential function of pluripotency factors in adult VSMCs.	Very low expression in adults; phenotype is often subtle; primarily used for developmental studies.	[107]
*Notch3*-KO	Directly and clearly reveals global effects on VSMC differentiation, maturation, and function.	Global knockout cannot distinguish cell-autonomous effects from systemic/compensatory influences.	[108]
*SM22α*-Cre	VSMC-specific Cre expression, enabling precise gene editing.	Minor leakiness (myeloid lineage/platelets); complex breeding and screening.	[109]
*Itga8*-CreER	Specifically targets arterial VSMCs, avoiding interference from visceral smooth muscle.	Leakiness in visceral smooth muscle; exhibits gender-specific effects.	[110]
*Myh11*-CreER	High specificity for mature VSMCs; suitable for lineage tracing.	Leakiness in pericytes; expression downregulated in advanced plaques.	[111]
*Acta2*-Cre/ER	Broad coverage of VSMCs across vascular beds; useful for studying phenotypic plasticity.	Leakiness in myofibroblasts; expression downregulated during phenotypic switching.	[112]

This table summarizes commonly used mouse strains for studying VSMC biology, including knockout models targeting key regulators (e.g., *Klf4*, *Notch3*) and Cre-based tools for VSMC-specific gene editing or lineage tracing (*SM22α-Cre*, *Myh11-CreER*, etc.). For each model, the advantages and limitations are highlighted to guide appropriate selection based on experimental context.

## 5. Therapeutic Strategies Targeting VSMC Plasticity

### 5.1 Direct Modulation of VSMC Phenotypes

Recent therapeutic approaches have aimed either to inhibit detrimental phenotypic switching in VSMCs or to promote their transition toward beneficial phenotypes [113,114,115]. Small-molecule agents and natural compounds capable of modulating key signaling pathways have demonstrated promising potential for clinical application [114]. For instance, recent studies have found that the small-molecule inhibitor CFI-402257, which targets TTK protein kinase, effectively suppresses the transition of VSMCs to a synthetic phenotype, reduces intimal hyperplasia and atherosclerosis progression, without interfering with vascular repair processes [116]. Similarly, Kanglexin (KLX), a novel anthraquinone derivative derived from traditional Chinese medicine, has been shown to directly bind and inhibit platelet-derived growth factor receptor-β (PDGFR-β). By blocking the downstream PDGFR-β–MEK–ERK–ELK-1/KLF4 signaling axis, KLX effectively prevents VSMC proliferation, migration, and dedifferentiation, thereby significantly attenuating atherosclerotic plaque formation and neointimal hyperplasia after balloon injury in animal models [117]. Compounds that enhance the expression of contractile genes or suppress pro-inflammatory signaling pathways also contribute to maintaining VSMCs in a plaque-stabilizing state [114,118]. Research indicates that targeting specific receptors, such as ChemR23, represents an effective strategy for modulating VSMC phenotypes. For example, the small-molecule agonist Chemerin-9 can maintain the contractile phenotype and suppress inflammation in VSMCs through activation of ChemR23, whereas loss of function of this receptor drives VSMCs toward a pro-atherogenic synthetic/macrophage-like phenotype. This provides novel evidence and insights for stabilizing plaques through precise intervention in signaling pathways [119].

Moreover, maintaining the stability of the VSMC cytoskeleton is crucial for phenotypic preservation. Studies have shown that cytoskeleton-associated genes *Fblim1*, *Tns1*, and *Synpo2* serve as key checkpoints for maintaining the contractile phenotype; their loss of function leads to cytoskeletal disorganization, driving phenotypic switching and exacerbating vascular remodeling. Recent studies further reveal that Tetraspanin 4 (TSPAN4) drives vascular VSMCs to switch from a contractile to a synthetic phenotype by downregulating Tropomyosin 1 (TPM1) and causing deterioration of the cytoskeleton, thereby promoting intimal hyperplasia [120]. This suggests that targeting cytoskeletal stability itself represents an emerging direction for preventing pathological switching of VSMCs [121].

Poly (ADP-ribose) polymerase 1 (PARP1) has been revealed as an upstream master regulator of VSMC phenotype [122,123,124]. It can modify key transcription factors to suppress the contractile phenotype while promoting proliferation and migration. Its inhibitors have shown therapeutic potential in animal models, establishing it as a novel interventional target [125]. Beyond targeting known protein entities, recent research is actively seeking to identify entirely new regulatory molecules as breakthrough points for therapeutic intervention. For example, recent studies discovered that the circRNA circSETD2(14,15) encodes a previously unknown hidden protein, p-414aa. This protein binds and inhibits the RNA-stabilizing factor HuR, thereby reducing the stability of pro-proliferative gene C-FOS mRNA and effectively suppressing VSMC phenotypic switching and neointimal hyperplasia. This finding not only reveals the crucial role of circRNA coding function in vascular remodeling but also establishes circSETD2(14,15) and its encoded product p-414aa as a novel class of potential therapeutic targets, expanding the dimension of drug development [126].

Epigenetic regulation, particularly the role of lncRNAs, has emerged as a new research direction in atherosclerosis treatment. Studies indicate that lncRNAs can precisely regulate vascular cell function and fate, and interventions targeting them offer advantages such as reversibility and high specificity [113,127]. In this context, specifically, the lncRNA CARMN modulates VSMC plasticity through interaction with SRF. Therefore, targeting this RNA holds promise for achieving precise regulation of VSMC behavior. Compared to conventional therapies, designing antisense oligonucleotides or small-molecule inhibitors to interfere with specific epigenetic regulators such as CARMN represents a concrete application of this strategy, enabling more targeted intervention with fewer side effects [49].

### 5.2 Immunotherapy Approaches

The immune-mediated precise clearance strategy targeting VSMCs represents an advanced therapeutic paradigm aimed at specifically eliminating pathogenic VSMC subsets within plaques through engineered immune modalities [128]. The core scientific foundation of this strategy lies in the pathological phenotypic switching exhibited by VSMCs during atherosclerosis progression, such as their transformation into synthetic, macrophage-like, or fibroblast-like phenotypes, which leads these cellular subsets to express specific surface antigens (e.g., FAP) [64,67]. By designing bispecific antibodies or chimeric antigen receptors capable of recognizing such antigens while simultaneously engaging immune effector cells (e.g., T cells), cytotoxic immune responses can be precisely directed against diseased VSMCs. This approach achieves the root-cause removal of key cells that drive plaque progression and instability, while avoiding damage to normal vascular wall cells [129,130,131]. This further reinforces the potential of immunotherapy as a novel treatment modality for atherosclerosis—that is, enabling the precise clearance of disease-driving cells through redirected or engineered immune cells [132].

This marks a shift in the therapeutic paradigm for atherosclerosis, moving from traditional “risk factor management” that primarily relies on lipid-lowering, toward interventions targeting the local cellular pathophysiology within plaques. Unlike conventional drugs that broadly inhibit cell proliferation, such targeted immune strategies can distinguish pathological cells from normal tissue and selectively clear specific cell types responsible for plaque progression and vulnerability, thereby improving therapeutic efficiency and reducing systemic side effects. However, the clinical translation of such strategies necessitates careful evaluation of on-target/off-tumor toxicity and the development of human-specific VSMC subtype markers.

### 5.3 Nanotechnology for Targeted Delivery

Advances in nanoparticle design have enabled the precise delivery of therapeutic agents to specific VSMCs subsets within atherosclerotic plaques. Functionally modified nanoparticles can be engineered as carriers that recognize surface markers on modulated VSMCs, thereby achieving localized release of drugs, nucleic acids, or imaging agents [133,134]. For example, Maiseyeu et al. designed gelatinase-activatable nanoparticles (GlpNPs) that display phosphatidylserine on their surface as an “eat-me” signal. These nanoparticles effectively target and accumulate in dedifferentiated VSMCs within atherosclerotic plaques and deliver a Glucagon-like peptide-1 (GLP-1) receptor agonist. In mouse models, this system significantly reduced plaque cholesterol and inflammation at an extremely low dose that did not induce systemic metabolic changes (weight-neutral) [135]. Micellar nanoparticles displaying Monocyte Chemoattractant Protein-1 (MCP-1) peptides can specifically target synthetic VSMCs expressing high levels of C-C Chemokine Receptor Type 2 (CCR2) in atherosclerotic lesions and efficiently deliver therapeutic miRNA-145. In animal models, this targeted system markedly reduced plaque burden and successfully reprogrammed pathogenic cells toward a protective phenotype, with no significant systemic toxicity observed [136,137,138]. Furthermore, macrophage membrane-camouflaged nanoliposomes, constructed using biomimetic technology, can efficiently deliver the Proprotein Convertase Subtilisin/Kexin Type 9 (PCSK9) inhibitor Evolocumab to plaque VSMCs. By inhibiting PCSK9, this approach successfully reverses VSMC phenotypic switching, suppresses their proliferation and migration, and significantly attenuates atherosclerotic lesions in mouse models [139]. Such targeted delivery strategies minimize systemic drug exposure, enhance therapeutic efficacy, and reduce side effects.

Nanoparticle-based drug delivery systems also facilitate combination therapies that can simultaneously address multiple facets of VSMC dysfunction. For instance, nanoparticles can co-deliver antiproliferative agents (to limit the expansion of synthetic VSMCs) and pro-differentiation factors (to promote the restoration of a contractile phenotype). In fact, studies have identified certain single compounds, such as the novel carbazole derivative CAB, which can concurrently inhibit VSMC proliferation and promote the stabilization of their contractile phenotype, providing a new molecular basis for combination therapy [140]. Given the complexity of regulating VSMC plasticity, such multifaceted, synergistic therapeutic approaches may prove more effective than interventions targeting a single pathway. As summarized in Table 3, a variety of emerging therapeutic approaches aimed at VSMC plasticity have been identified.

**Table 3.** **Emerging therapeutic strategies targeting VSMC plasticity**.

Strategy	Mechanism of action	Development stage	Potential advantages
FAP-directed BiTE immunotherapy	Redirects T-cells to eliminate FAP+ modulated VSMCs	Preclinical validation	Lipid-independent, highly specific
Senolytic agents	Clears senescent VSMCs that impede vascular repair	Experimental models	Addresses aging-related pathology
CARMN lncRNA targeting	Modulates SRF-dependent VSMC gene expression	Early experimental	Epigenetic precision
Phenotype-specific nanoparticles	Targeted delivery to specific VSMC subsets	Design phase	Reduced off-target effects

This table summarizes representative strategies that modulate VSMC phenotype beyond traditional signaling pathways. For each approach, the mechanism of action, current stage of development, and potential advantages are outlined. These strategies collectively reflect a paradigm shift toward more precise, context-specific interventions, ranging from epigenetic modulation to cell-selective clearance and targeted delivery in the treatment of atherosclerosis. FAP, fibroblast activation protein; BiTE, bispecific T-cell engager.

### 5.4 Imaging and Diagnostic Innovations

Advanced imaging techniques capable of specifically visualizing the phenotypes of VSMCs within plaques are crucial for both disease diagnosis and treatment monitoring. For instance, PET integrated with magnetic resonance imaging (MRI), based on the Cre/lox system, has successfully enabled the non-invasive visualization of plaque areas derived from smooth muscle cells in mouse models [141]. PET ligands targeting FAP allow for the non-invasive detection of modulated VSMCs in human atherosclerotic lesions [5,142,143]. Similarly, MRI and computed tomography (CT) techniques can identify plaque features associated with VSMCs, such as fibrous cap integrity and microcalcification patterns, providing valuable information for prognostic assessment [141,144]. Furthermore, multimodal nanoprobes targeting other plaque-associated molecules, such as the inflammatory marker CD40, offer novel approaches for identifying high-risk plaques through modalities like MRI [145].

Progress in these imaging technologies has facilitated the development of precision medicine approaches, enabling clinicians to stratify patients based on plaque composition rather than relying solely on the degree of luminal stenosis. Patients with plaques rich in pathological VSMCs phenotypes may receive more aggressive targeted therapies, while those with stable plaques can avoid unnecessary interventions.

## 6. Future Research Directions and Challenges

**Fig. 2.**
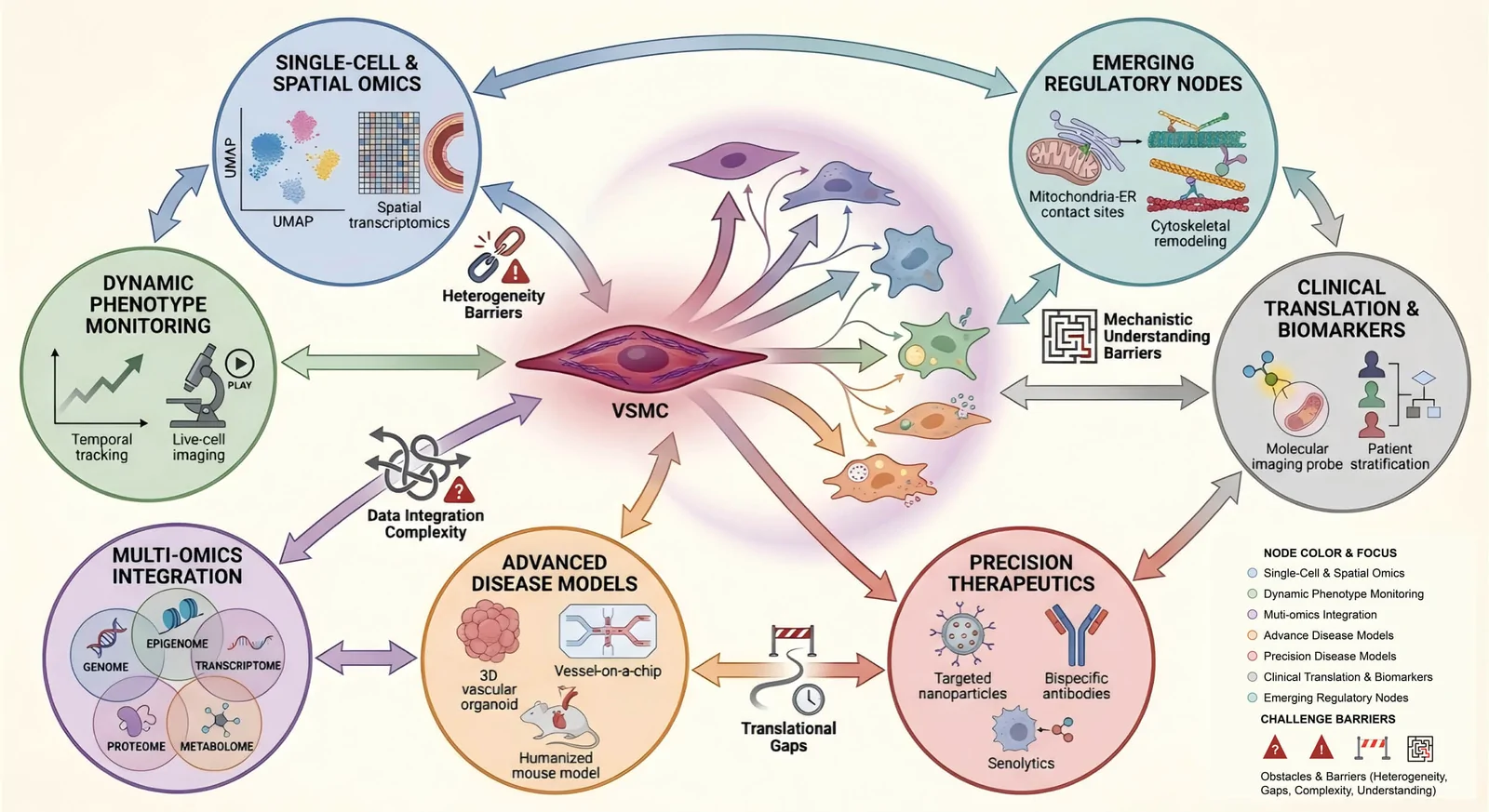
**VSMC phenotypic regulation & clinical translation**. This figure provides a comprehensive overview of the cutting-edge directions, key technologies, and translational pathways in research on VSMC plasticity. The left side highlights the central role of single-cell and spatial omics technologies (e.g., scRNA-seq, spatial transcriptomics) in dynamic phenotype monitoring and multi-omics data integration, revealing the high heterogeneity of VSMCs. The right side focuses on emerging regulatory nodes (e.g., mitochondria-ER contact sites, cytoskeletal remodeling) and precision therapeutic strategies (including targeted nanoparticles, bispecific antibodies, and senolytics). At the core, VSMC plasticity and heterogeneity serve as the central hub linking mechanistic investigation, technological advances, and clinical translation, surrounded by key challenges (such as heterogeneity, mechanistic complexity, and translational barriers).

While significant progress has been made in understanding the plasticity of VSMCs in atherosclerosis, translating these insights into effective therapeutic strategies requires overcoming a series of interconnected scientific challenges [71]. Future research must shift towards a more integrated and dynamic paradigm, focusing on several core, intersecting domains (Fig. 2).

Investigations must move beyond static molecular snapshots toward a systems-level understanding of the dynamic regulatory networks governing phenotypic switching. This necessitates elucidating the real-time interplay among epigenetic reprogramming, metabolic alterations, and signaling pathways across different stages of the disease. Employing integrated multi-omics analyses, real-time live-cell imaging, and precise gene-editing technologies is crucial for deciphering the temporal logic underlying VSMCs fate decisions. Furthermore, exploring the functions of emerging regulatory nodes, such as mitochondria-endoplasmic reticulum communication and cytoskeletal remodeling, may reveal novel targets for therapeutic intervention.

Although single-cell technologies have confirmed the high degree of VSMCs heterogeneity [64], the three-dimensional spatial distribution of distinct subpopulations, their intercellular communication networks, and their specific contributions to plaque stability remain poorly defined. Future studies should leverage high-resolution spatial multi-omics techniques and employ advanced models such as organoids and vessel-on-a-chip systems to simulate the plaque microenvironment. This will enable a clearer elucidation of the dynamic crosstalk between VSMCs, immune cells, and the extracellular matrix, thereby allowing precise correlation of cellular states with pathological outcomes.

In addition, existing animal models based on the Cre-loxP system still exhibit limitations in cell-type specificity, leaky expression, and their ability to faithfully recapitulate human disease pathophysiology. There is a pressing need to develop next-generation, spatiotemporally controllable gene-editing models and humanized mouse models to better simulate the progression of human atherosclerosis. Concurrently, the integration of longitudinal monitoring via non-invasive imaging techniques (e.g., FAP-PET) with artificial intelligence and computational modeling for the analysis and integration of multidimensional data will facilitate a predictive understanding of VSMCs behavior and drug responses.

To achieve precise clinical translation, it is essential to prioritize multicenter cohort studies that utilize human samples to validate key mechanisms identified in animal models and to establish robust correlations between VSMC phenotypes and clinical outcomes. Building on this foundation, the accelerated development and translation of novel therapies targeting specific VSMC subpopulations, such as bispecific antibodies, epigenetic modulators, anti-aging therapies (e.g., senolytics/senomorphics), and intelligent nanodelivery systems from preclinical research to early-phase clinical trials is imperative. Optimizing their specificity, safety, and delivery efficiency will be key to realizing the ultimate therapeutic goal of plaque stabilization and improved patient prognosis.

## 7. Conclusion

VSMC plasticity is a cornerstone of atherosclerotic pathogenesis, governing disease progression and clinical outcomes. Traditionally viewed as terminally differentiated, VSMCs can, in fact, transition into diverse functional states under the influence of complex inflammatory, metabolic, and mechanical signals, participating in plaque formation, stabilization, and rupture. Recent breakthroughs in single-cell technologies and lineage-tracing studies have not only unveiled the high heterogeneity and clonal expansion characteristics of VSMCs within plaques but also provided novel insights into their roles in immune regulation, cellular senescence, and tissue remodeling.

On the therapeutic front, intervention strategies targeting VSMC plasticity are evolving from traditional non-specific anti-proliferative approaches toward new directions, such as precisely targeting pathological subpopulations, modulating epigenetic states, clearing senescent cells, and employing intelligent drug delivery systems. These strategies aim to restore a stable VSMC phenotype, inhibit detrimental transitions, thereby promoting plaque stability and reducing the risk of cardiovascular events.

Looking ahead, key future directions for atherosclerosis research include further elucidating the dynamic regulatory networks governing VSMC plasticity, deciphering its authentic behavior in humans, and developing specific and safe targeted therapies. Through interdisciplinary collaboration and technological innovation, we can anticipate a more precise understanding and more effective treatment of this disease, ultimately improving clinical outcomes for cardiovascular disease patients.

## Abbreviations

VSMCs, Vascular smooth muscle cells; ECM, Extracellular matrix; PDGF, Platelet-derived growth factor; TGF-β, Transforming growth factor-β; MMP-2, Matrix metalloproteinase-2; SMAD, Small mothers against decapentaplegic; AGGF1, Angiogenic factor with G patch and FHA domain 1; EndMT, Endothelial-to-mesenchymal transition; KLF4, Krüppel-like factor 4; SRF, Serum response factor; NRP1, Neuropilin 1; ITGB3, Integrin subunit beta 3; RBM24, RNA-binding motif protein 24; JAK2, Janus kinase 2; STAT3, Signal transducer and activator of transcription 3; NEXN, Nexilin; ATF4, Activating transcription factor 4; SUV39H1, Suppressor of variegation 3-9 homolog 1; ELK1, ETS Like-1 protein; SENP1, SUMO-specific protease 1; KAT2B, Lysine acetyltransferase 2B; MYOCD, Myocardin; lncRNA, Long non-coding RNA; miRNA, microRNA; MAPK, Mitogen-activated protein kinase; ERK, Extracellular signal-regulated kinase; PHB2, Prohibitin 2; hnRNPA1, Heterogeneous nuclear ribonucleoprotein A1; PKM2, Pyruvate kinase M2; PI3K, Phosphatidylinositol 3-kinase; AKT, Protein kinase B; mTOR, Mammalian target of rapamycin; CBP, CREB-binding protein; TET2, Ten-eleven translocation 2; HDACs, Histone deacetylases; SASP, Senescence-associated secretory phenotype; JNK, c-Jun N-terminal kinase; BMP6, Bone morphogenetic protein 6; IL-1β, Interleukin 1-β; TNF-α, Tumor necrosis factor α; RUNX2, Runt-related transcription factor 2; EDIL3, EGF-like repeats and discoidin I-like domains 3; CCL19, C-C motif chemokine ligand 19; CCL21, C-C motif chemokine ligand 21; CXCL13, C-X-C motif chemokine ligand 13; ox-LDL, Oxidized low-density lipoprotein; ATLOs, Artery tertiary lymphoid organs; PTLOs, Plaque tertiary lymphoid organs; scRNA-seq, Single-cell RNA sequencing; FAP, Fibroblast activation protein; PET, Positron emission tomography; PCR, Polymerase chain reaction; SCA1, Smooth muscle cell-specific antigen 1; PROTAC, Proteolysis-targeting chimera; TRAP1, TNF receptor-associated protein 1; NR4A3, Nuclear receptor subfamily 4 group A member 3; SIRT6, Sirtuin 6; CHIP, Carboxyl terminus of Hsc70-interacting protein; STING, Stimulator of interferon genes; IRF3, Interferon regulatory factor 3; C/EBPα, CCAAT/enhancer-binding protein alpha; DPP4, Dipeptidyl Peptidase 4; SOX9, SRY-box transcription factor 9; PARP1, Poly (ADP-ribose) polymerase 1; circRNA, circular RNA; GLP-1, Glucagon-like peptide-1; MCP-1, Chemoattractant Protein-1; CCR2, C-C Chemokine Receptor Type 2; PCSK9, Proprotein Convertase Subtilisin/Kexin Type 9; MRI, Magnetic resonance imaging; CT, Computed tomography.
